# Betulinic Acid Suppresses Ovarian Cancer Cell Proliferation through Induction of Apoptosis

**DOI:** 10.3390/biom9070257

**Published:** 2019-07-03

**Authors:** Dahae Lee, Seoung Rak Lee, Ki Sung Kang, Yuri Ko, Changhyun Pang, Noriko Yamabe, Ki Hyun Kim

**Affiliations:** 1 School of Pharmacy, Sungkyunkwan University, Suwon 16419, Korea; 2 College of Korean Medicine, Gachon University, Seongnam 13120, Korea; 3 Department of Obstetrics and Gynecology, University of Ulsan, Asan Medical Center, Seoul 05505, Korea; 4 School of Chemical Engineering, Sungkyunkwan University, Suwon 16419, Korea

**Keywords:** *Cornus walteri*, betulinic acid, ovarian cancer, apoptosis, mitochondria-dependent pathway, mitochondria-independent pathway

## Abstract

Ovarian cancer is one of the leading causes of cancer deaths worldwide in women, and the most malignant cancer among the different gynecological cancers. In this study, we explored potentially anticancer compounds from *Cornus walteri* (Cornaceae), the MeOH extract of which has been reported to show considerable cytotoxicity against several cancer cell lines. Phytochemical investigations of the MeOH extract of the stem and stem bark of *C. walteri* by extensive application of chromatographic techniques resulted in the isolation of 14 compounds (**1**–**14**). The isolated compounds were evaluated for inhibitory effects on the viability of A2780 human ovarian carcinoma cells and the underlying molecular mechanisms were investigated. An 3-(4,5-dimethylthiazol-2-yl)-2,5-diphenyltetrazolium bromide (MTT) assay was employed to assess the anticancer effects of compounds **1**–**14** on A2780 cells, which showed that compound **11** (betulinic acid) reduced the viability of these cells in a concentration-dependent manner and had an half maximal (50%) inhibitory concentration (IC_50_) of 44.47 μM at 24 h. Nuclear staining and image-based cytometric assay were carried out to detect the induction of apoptosis by betulinic acid. Betulinic acid significantly increased the condensation of nuclei and the percentage of apoptotic cells in a concentration-dependent manner in A2780 cells. Western blot analysis was performed to investigate the underlying mechanism of apoptosis. The results indicated that the expression levels of cleaved caspase-8, -3, -9, and Bax were increased in A2780 cells treated with betulinic acid, whereas those of Bcl-2 were decreased. Thus, we provide the experimental evidence that betulinic acid can induce apoptosis in A2780 cells through both mitochondria-dependent and -independent pathways and suggest the potential use of betulinic acid in the development of novel chemotherapeutics for ovarian cancer therapy.

## 1. Introduction

**Figure 1:**
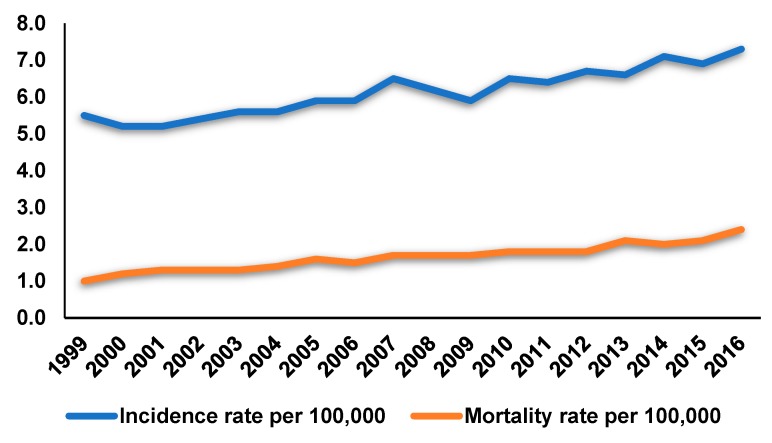
Age-standardized incidence rates and mortality rates per 100,000 for ovarian cancer between 1999 and 2016 in the Korea Central Cancer Registry.

Ovarian cancer is one of the leading causes of cancer deaths worldwide in women. It is estimated that in the United States there were 14,070 ovarian cancer deaths and 22,240 new cases of ovarian cancer diagnosed in 2018 [1]. In the United States, the overall rate of incidence of ovarian cancer declined from 1985 to 2014 and the mortality rates also exhibited a steady downward tendency between 1976 and 2015. However, the situation regarding ovarian cancer in Korea is rather different. Among the three major gynecologic cancers, namely cervical, endometrial, and ovarian cancers, the age-standardized incidence rate of cervical cancer in Korea decreased between 1999 and 2016, whereas that of endometrial and ovarian cancers gradually increased during this period (Figure 1), as per the data from the Korea Central Cancer Registry. Particularly, the mortality rates of ovarian cancer between 1999 and 2016 in Korea also increased (Figure 1). Ovarian cancer is the most malignant cancer among the different gynecological cancers. Despite recent advances in the standard treatment methods for ovarian cancer, including chemotherapy and surgery [2], the overall five-year survival rate for all ovarian cancer patients remains about 30%, with a fairly poor prognosis [3]. Therefore, attention has been focused on searching for novel anti-tumor therapeutics for ovarian cancer. Natural products, which have several advantages, such as efficiency and minimal side effects, have garnered increasing attention to be developed as anti-tumor therapeutics [4]. Alternative therapeutic approaches for cancer treatment are also urgently needed considering the resistance developed in cancers against a vast majority of clinically established anticancer drugs. Therefore, implementation of rational alternative medication using anti-tumoral natural products could be one of the choices to treat ovarian cancer and to overcome drug resistance.

**Figure 2:**
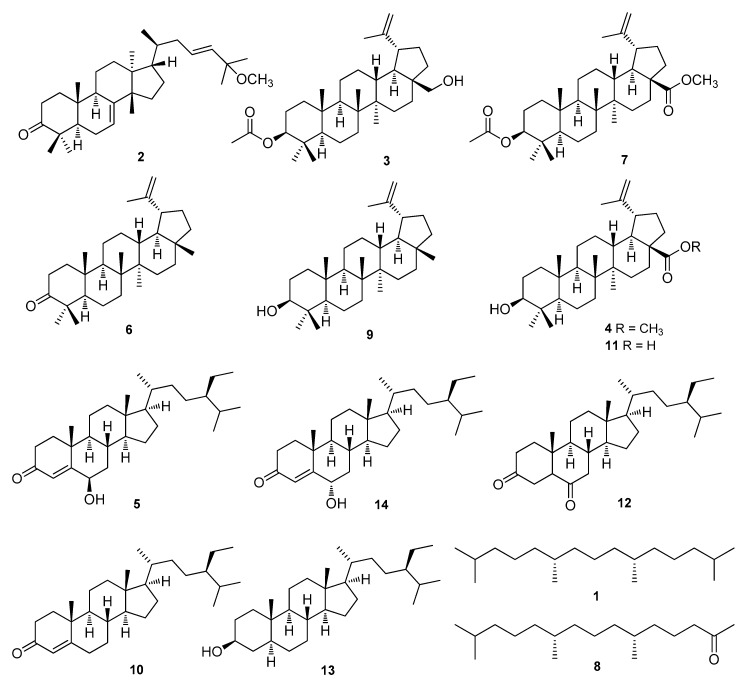
The chemical structures of compounds **1**–**14**.

*Cornus walteri* Wagner (Cornaceae) is a deciduous tree that is widely distributed in the valley regions of Korea and East Asia. The fruits and leaves of *C. walteri* have been mainly used as a traditional medicine in China for the treatment of glycosuria and skin inflammatory symptoms [5]. In Korean folk medicine, the leaves of this plant are utilized for alleviation of diarrheal symptoms [6]. In a previous study on this natural source, anti-inflammatory, anti-obesity, anti-photoaging, and antihyperglycemic activities were reported from crude extracts of *C. walteri* [7,8]. As part of our ongoing endeavor to find structurally and/or biologically novel natural products from a variety of natural resources [9,10,11,12,13], we found that MeOH extract of stem and stem bark of *C. walteri* showed considerable cytotoxicity against several cancer cell lines, including A549, SK-OV-3, and SK-MEL-2 [14], which led to the phytochemical investigation on the MeOH extract to identify the bioactive compounds responsible for the observed effects. Chemical analysis of the MeOH extract by our group led to the identification of cytotoxic compounds, including *δ*-valerolactones, tirucallane, and lupane triterpenoids [14,15]. Inspired by the identification of cytotoxic compounds, we further investigated the MeOH extract of the stem and stem bark of *C. walteri* to identify cytotoxic phytochemicals, by the application of extensive chromatographic techniques. These subsequent studies resulted in the successful isolation of 14 compounds (**1**–**14**) (Figure 2). Herein, we describe the isolation and structural elucidation of all the isolates (**1**–**14**) as well as the investigation of the molecular mechanism of the anti-cancer actions of the isolates in a human ovarian carcinoma line.

## 2. Materials and Methods

### 2.1. General Experimental Procedures

Optical rotations were calculated using a Jasco P-1020 polarimeter (Jasco, Easton, MD, USA). Infra-red (IR) spectra were obtained using a Bruker IFS-66/S FT-IR spectrometer (Bruker, Karlsruhe, Germany). Electrospray ionization (ESI) mass spectra were obtained using a Waters Micromass Q-Tof Ultima electrospray ionization time-of-flight (ESI-TOF) mass spectrometer (Waters, New York, NY, USA). NMR spectra were recorded on a Varian UNITY INOVA 500 NMR spectrometer operating at 500 MHz (^1^H) and 125 MHz (^13^C) with chemical shifts (δ) given in ppm. Preparative high-performance liquid chromatography (HPLC) was performed using a Gilson 306 pump with a Shodex refractive index detector. Semi-preparative HPLC was performed utilizing a Shimadzu Prominence HPLC system with SPD-20A/20AV UV-Vis detectors (Shimadzu, Tokyo, Japan). LC/MS analysis was conducted on an Agilent 1200 Series HPLC system (Agilent Technologies, Santa Clara, CA, USA) using an analytical Kinetex column (4.6 × 100 mm, 3.5 μm) followed by a 6130 Series ESI mass spectrometer equipped with a diode array detector. Silica gel 60 (Merck, 70-230 mesh and 230-400 mesh) and RP-C_18_ silica gel (Merck, 40–63 μm) were used for column chromatography. The packing material for molecular sieve column chromatography was Sephadex LH-20 (Pharmacia, Uppsala, Sweden). Merck precoated silica gel F_254_ plates and RP-18 F_254s_ plates (Merck, Darmstadt, Germany) were used for thin-layer chromatography (TLC). Spots were detected on the TLC plates either under UV light or by heating after spraying with anisaldehyde-sulfuric acid.

### 2.2. Plant Material

*C. walteri* stems and stem bark were obtained from Jeju Island, Korea, in October 2014. The plants were identified by one of the authors (K. H. K). A voucher specimen (SKKU MC-2014) was deposited in the herbarium of the School of Pharmacy, Sungkyunkwan University, Suwon, Korea.

### 2.3. Extraction and Isolation

The stems and stem bark of *C. walteri* (3.0 kg) were dried, chopped, extracted with 80% MeOH (5 L × 3) at room temperature, and then filtered. The crude MeOH extract was concentrated in vacuo to obtain a MeOH extract (310 g). The MeOH extract was dissolved in distilled water (6.5 L) and successively solvent-partitioned using hexane, CHCl_3_, and *n*-BuOH (700 mL × 3), which resulted in 15.0, 32.0, and 45.0 g of each fraction, respectively. The hexane-soluble fraction (15.0 g) was fractionated by silica gel column chromatography and eluted with a gradient solvent system of hexane-EtOAc (5:1 to 1:1, *v*/*v*) to obtain five fractions (H1–H5). Fraction H1 (4.0 g) was separated by an RP-C_18_ silica gel column using 100% MeOH to acquire five fractions (H11–H15). Fraction H13 (800 mg) was further separated by silica gel column chromatography eluting a gradient solvent system of hexane-EtOAc (7:1 to 1:1, *v*/*v*) to provide six subfractions (H131–H136). Compound **14** (6 mg) was purified from subfraction H131 (100 mg) by semi-preparative normal-phase HPLC (Apollo Silica column, 250 × 10.0 mm, 5 μm, flow rate: 2 mL/min) and eluted with an isocratic solvent system of hexane-EtOAc (18:1, *v*/*v*). Five subfractions (H141-H145) were acquired from fraction H14 (300 mg) by silica gel column chromatography with a gradient solvent system of hexane-EtOAc (16:1 to 1:1, *v*/*v*). Subfraction H142 (50 mg) was isolated by semi-preparative normal-phase HPLC (Apollo Silica column, 250 × 10.0 mm, 5 μm, flow rate: 2 mL/min) with an isocratic solvent system of hexane-EtOAc (28:1, *v*/*v*) to afford compound **3** (40 mg). Compound **1** (30 mg) was obtained from subfraction H144 (50 mg) by semi-preparative normal-phase HPLC (Apollo Silica column, 250 × 10.0 mm, 5 μm, flow rate: 2 mL/min) eluted with an isocratic solvent system of hexane-EtOAc (12:1, *v*/*v*). Fraction H15 (100 mg) was separated by a C18 Sep-Pak column eluting an isocratic solvent system of hexane-EtOAc (10:1, *v*/*v*) and further isolated by utilizing semi-preparative normal-phase HPLC (Apollo Silica column, 250 × 10.0 mm, 5 μm, flow rate: 2 mL/min) with an isocratic solvent system of hexane-EtOAc (20:1, *v*/*v*) to afford compound **4** (7 mg). Fraction H2 (2.5 g) was loaded onto an RP-C_18_ silica gel column and separated by eluting with 100% MeOH to obtain eight fractions (H21-H28). Compound **5** (60 mg) was isolated from fraction H27 (150 mg) by utilizing semi-preparative normal-phase HPLC (Apollo Silica column, 250 × 10.0 mm, 5 μm, flow rate: 2 mL/min) with an isocratic solvent system of hexane-EtOAc (8:1, *v*/*v*). Fraction H28 (100 mg) was isolated by semi-preparative normal-phase HPLC (Apollo Silica column, 250 × 10.0 mm, 5 μm, flow rate: 2 mL/min) with an isocratic solvent system of hexane-EtOAc (8:1, *v*/*v*) to obtain compound **11** (25 mg). Fraction H3 (1.7 g) was loaded onto an RP-C_18_ silica gel column and fractionated by eluting with 100% MeOH to obtain six fractions (H31–H36). Fraction H32 (200 mg) was separated by reverse-phase Lobar column with an isocratic solvent system of hexane-EtOAc (4:1, *v*/*v*) to obtain four subfractions (H321–H324). Compound **7** (4 mg) was obtained from subfraction H321 (150 mg) by semi-preparative reverse-phase HPLC (Econosil C18 column, 250 × 10.0 mm, 5 μm, flow rate: 2 mL/min) eluting with an isocratic solvent system of 95% MeOH. Fraction H33 (400 mg) was fractionated by reverse-phase Lobar column with an isocratic solvent system of hexane-EtOAc (4:1, *v*/*v*) to obtain five subfractions (H331–H335). Fraction H331 (50 mg) was purified by semi-preparative reverse-phase HPLC (Econosil C18 column, 250 × 10.0 mm, 5 μm, flow rate: 2 mL/min) with an isocratic solvent system of 100% MeOH to give compound **2** (7 mg). Compound **8** (30 mg) was obtained from fraction H34 (120 mg) by semi-preparative normal-phase HPLC (Apollo Silica column, 250 × 10.0 mm, 5 μm, flow rate: 2 mL/min) with an isocratic solvent system of hexane-EtOAc (3:1, *v*/*v*). Compounds **10** (5 mg) and **12** (8 mg) were purified from fraction H36 (120 mg) by semi-preparative normal-phase HPLC (Apollo Silica column, 250 × 10.0 mm, 5 μm, flow rate: 2 mL/min) with an isocratic solvent system of hexane-EtOAc (4:1, *v*/*v*). Fraction H4 (1.3 g) was loaded onto an RP-C_18_ silica gel column and fractionated by eluting with 100% MeOH to give six fractions (H41–H46). Fraction H44 (120 mg) was isolated by semi-preparative normal-phase HPLC (Apollo Silica column, 250 × 10.0 mm, 5 μm, flow rate: 2 mL/min) with an isocratic solvent system of hexane-EtOAc (2:1, *v*/*v*) to obtain compounds **6** (5 mg) and **9** (25 mg). Fraction H5 (500 mg) was fractionated using an RP-C_18_ silica gel column with an isocratic solvent system of 85% MeOH to obtain five fractions (H51–H55). Fraction H51 (150 mg) was passed over Sephadex-LH20 column with CHCl_3_-MeOH (1:1, *v*/*v*) and further purified by semi-preparative reverse-phase HPLC (Econosil C_18_ column, 250 × 10.0 mm, 5 μm, flow rate: 2 mL/min) eluting with an isocratic solvent system of 100% MeOH to obtain compound **13** (6 mg).

### 2.4. Cell Culture

The A2780 human ovarian cancer cell line was purchased from the American Type Culture Collection (Rockville, MD, USA) and cultured in Roswell Park Memorial Institute (RPMI) 1640 (Cellgro, Manassas, VA, USA), containing 10% fetal bovine serum (FBS), 1% penicillin/streptomycin, and 4 mM L-glutamine, at 37 °C under an atmosphere of 5% carbon dioxide and 95% air.

### 2.5. 3-(4,5-Dimethylthiazol-2-yl)-2,5-diphenyltetrazolium bromide (MTT) Cell Viability Assay

The cells were plated into 96-well culture plates at a density of 1 × 10^4^ cells per well and were allowed to adhere for 24 h. Thereafter, cells were either treated with 0.5% DMSO (control), or the indicated concentrations of compounds dissolved in 0.5% DMSO for 24 h and incubated with Ez-Cytox reagent kit (Daeil Lab Service, Seoul, Korea), according to the manufacturer’s instruction for 2 h at 37 °C. Cell viability was determined by measuring the absorbance of the wells (with cells) and the blank wells (without cells) at 450 nm using a microplate reader (PowerWave XS; Bio-Tek Instruments, Winooski, VT, USA).

### 2.6. Cell Staining with Hoechst 33342

The cells were plated into 6-well culture plates at a density of 4 × 10^5^ cells per well and allowed to adhere for 24 h. Thereafter, cells were treated with 25 and 50 μM of compound **11** for 24 h and stained with Hoechst 33342 solution (Thermo Fisher Scientific, Waltham, MA, USA) for 10 min. Fluorescent images were obtained using an IX50 fluorescent microscope Olympus, Tokyo, Japan) equipped with a charge coupled device (CCD) camera.

### 2.7. Image-Based Cytometric Assay

To detect the apoptotic cell population, the cells were plated into 6-well culture plates at a density of 4 × 10^5^ cells per well and were allowed to adhere for 24 h. Thereafter, cells were treated with 25 and 50 μM of compound **11** for 24 h and stained with annexin V-Alexa Fluor 488, according to the manufacturer’s instructions for the image-based cytometric assay kit (Invitrogen, Carlsbad, CA, USA). After incubation at room temperature for 30 min in the dark, the percentage of apoptotic cells was calculated using a Tali image-based cytometer (Invitrogen, Temecula, CA, USA).

### 2.8. Western Blot Analysis

The cells were plated into 6-well culture plates at a density of 4 × 10^5^ cells per well and were allowed to adhere for 24 h. Thereafter, cells were treated with 25 and 50 μM of compound **11** for 24 h and floating cells present in the culture medium were harvested with a scraper. After centrifugation, the cell pellet was lysed with radioimmunoprecipitation assay (RIPA) buffer (Cell Signaling Technology, MA, USA), supplemented with 1 mM phenylmethylsulfonyl fluoride, on ice, according to the manufacturer’s instructions. After quantification of protein content using bicinchoninic acid (BCA) protein assay kit (Thermo Fisher Scientific, Waltham, MA, USA), equal amounts of proteins (20 µg/lane) were separated by electrophoresis through a precast 4%–15% Mini-PROTEAN TGX gel (Bio-Rad, Hercules, CA, USA) and transferred to a polyvinylidene difluoride membrane (Merck Millipore, Darmstadt, Germany). The membranes were probed with the epitope-specific primary and secondary antibodies and then visualized using ECL Advance Western Blotting Detection Reagents (GE Healthcare, Cambridge, UK) and a FUSION Solo Chemiluminescence System (PEQLAB Biotechnologie GmbH, Erlangen, Germany).

### 2.9. Statistical Analysis

Statistical significance was determined using analysis of variance (ANOVA) followed by a multiple comparison test with Bonferroni’s adjustment. A *p*-value less than 0.05 was considered to be statistically significant.

## 3. Results and Discussions

### 3.1. Isolation and Identification of Compounds ***1**–**14***

We explored the cytotoxic phytochemicals present in the MeOH extract of the stem and stem bark of *C. walteri* because the extract showed considerable cytotoxicity against several cancer cell lines in our previous work [14]. The MeOH extract was solvent-partitioned with hexane, CHCl_3_, *n*-BuOH, and water, and significant spots were detected in the hexane-soluble fraction on the basis of TLC analysis. Thus, the hexane-soluble fraction was subjected to repeated column chromatography and preparative HPLC isolation, which afforded the isolation of 14 compounds, including seven triterpenoids (**2**–**4**, **6**, **7**, **9**, **11**), five steroids (**5**, **10**, **12**–**14**), and two diterpene analogs (**1** and **8**) (Figure 2). The chemical structures of all the isolates were determined and they were identified as norphytan (**1**) [16], leucophyllone (**2**) [14], 3-*O*-acetylbetulin (**3**) [17], betulinic acid methyl ester (**4**) [18], 6*β*-hydroxysitostenone (**5**) [19], lupenone (**6**) [20], methyl 3-*O*-acetylbetulinate (**7**) [21], phytone (**8**) [22], lupeol (**9**) [23], sitostenone (**10**) [24], betulinic acid (**11**) [25], 5*α*-stigmast-3,6-dione (**12**) [26], 3*β*-sitostanol (**13**) [27], and 6*α*-hydroxy-*β*-sitostenone (**14**) [28] by comparison of their spectroscopic data, including ^1^H and ^13^C NMR (see the Supplementary Materials), and physical data with previously reported values, and LC/MS analysis. Compounds **1**, **5**, **8**, **10**, and **12–14** were isolated and identified for the first time from *C. walteri*.

### 3.2. Cytotoxic Effects of the Isolated Compounds ***1**–**14*** on A2780 Human Ovarian Carcinoma Cells

**Figure 3:**
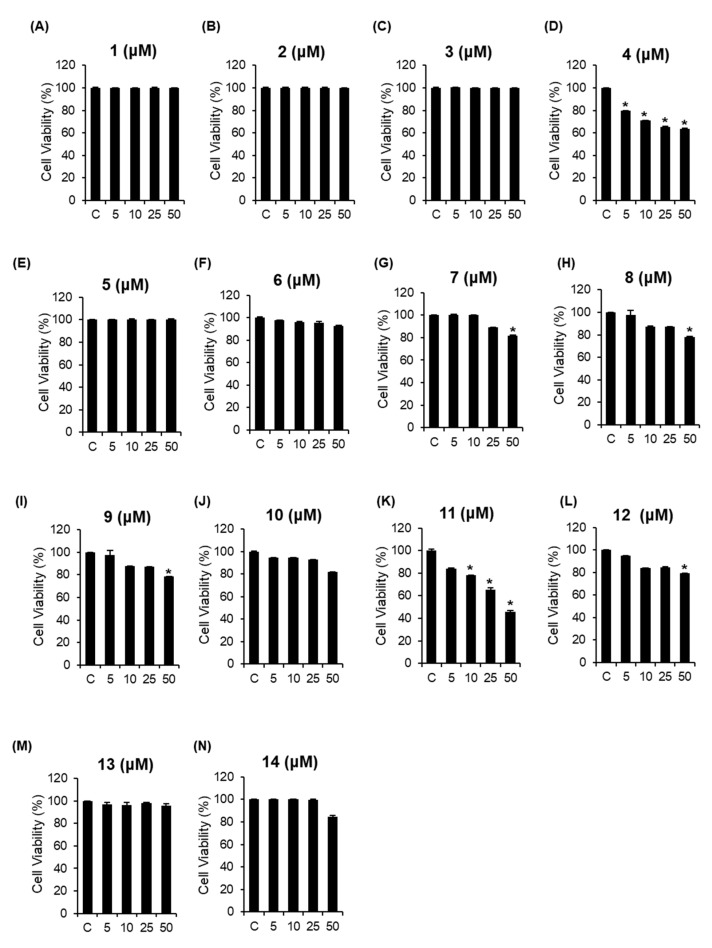
Comparison of the inhibitory effects of the isolated compounds **1–14** (**A–N**) on A2780 human ovarian carcinoma cells. * *p* < 0.05 compared to the value obtained for non-treated cells. Cell viability assays were done in triplicate for each assay and repeated at least thrice.

We investigated whether the isolated compounds **1–14** could inhibit the viability of A2780 human ovarian carcinoma cells. The MTT cell viability assays were performed with compounds **1**–**14** to determine their cytotoxic effects on A2780 cells [29,30,31,32]. Of the tested compounds, compounds **4** and **11** significantly suppressed cell proliferation in a concentration-dependent manner and compound **11,** particularly, showed the strongest effect, with an IC_50_ value of 44.47 ± 0.01 μM, whereas compounds **7**, **8**, **9**, and **12** weakly reduced the viability of A2780 cells at 50 μM. The rest of the compounds showed no efficacy against the proliferation of these cells (Figure 3). Despite having the same triterpene skeleton, a difference in the relative cytotoxicity between compounds **4** and **11** was observed, suggesting that the presence of the methoxy group in COOH might be responsible for the decrease in potency. These results demonstrated that compound **11** would have the highest inhibitory activity against the viability of A2780 cells, which led to the verification of the molecular mechanism of action of compound **11** (betulinic acid).

### 3.3. Effects of Compound ***11*** on Apoptotic Death of A2780 Cells

**Figure 4:**
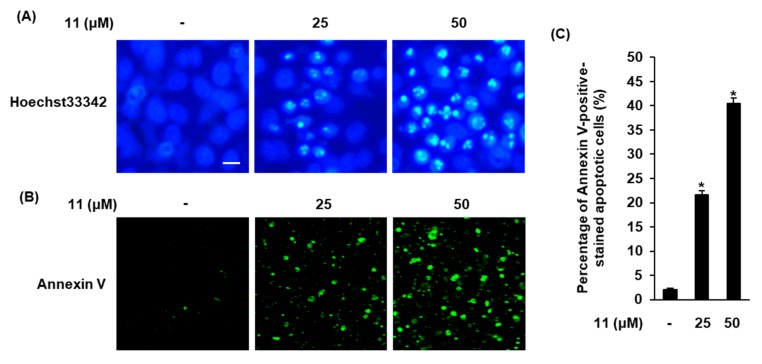
Effects of compound **11** on apoptotic death of A2780 cells. (**A**) Visualization of the condensation of nuclei stained with Hoechst 33342. Scale bar, 50 μm. (**B**) Visualization of apoptotic cells stained with annexin V (40× magnification). (**C**) Bar graph representing percentage of apoptotic cells. * *p* < 0.05 compared to the value obtained for non-treated cells. Image-based cytometric assays were done in triplicate and repeated at least three times.

Apoptosis is a form of programmed cell death and a major cancer hallmark that is characterized by typical biochemical features, including cellular shrinkage, DNA fragmentation, membrane blebbing, and chromatin condensation [33]. In the present study, cells treated with compound **11** were stained with Hoechst 33342 to determine the alterations in the nuclear morphology of A2780 cells. Slight blue fluorescence was observed in non-treated cells (control), whereas A2780 cells treated with compound **11** showed enhancement in Hoechst 33342 staining in a concentration-dependent manner. Cells treated with 50 μM of compound **11** were seriously damaged and showed bright blue Hoechst 33342-stained condensed chromatin of apoptotic cells (Figure 4A). These changes in the nuclear morphology of cells suggest that compound **11** induces apoptotic cell death. The percentage of apoptotic cells was measured by image-based cytometric assay. As shown in Figure 4B, the percentage of apoptotic cells stained with annexin V, which detects phosphatidylserine on the external membrane of apoptotic cells, increased significantly in a concentration-dependent manner after treatment with compound **11**. The percentage of non-treated cells (control) was 2.13% ± 0.25%; it increased to 21.63% ± 0.80% and 40.46% ± 1.15% when the concentration of compound **11** increased from 25 µM to 50 µM, respectively (Figure 4C). These results implied that compound **11** could significantly increase the condensation of nuclear and apoptotic cells, indicating that the apoptotic response was markedly potentiated after treatment of A2780 cells with compound **11**. These results indicated that compound **11** could induce apoptosis in A2780 cells.

### 3.4. Effects of Compound ***11*** on the Expression of Apoptosis-Related Proteins in A2780 Human Ovarian Carcinoma Cells

**Figure 5:**
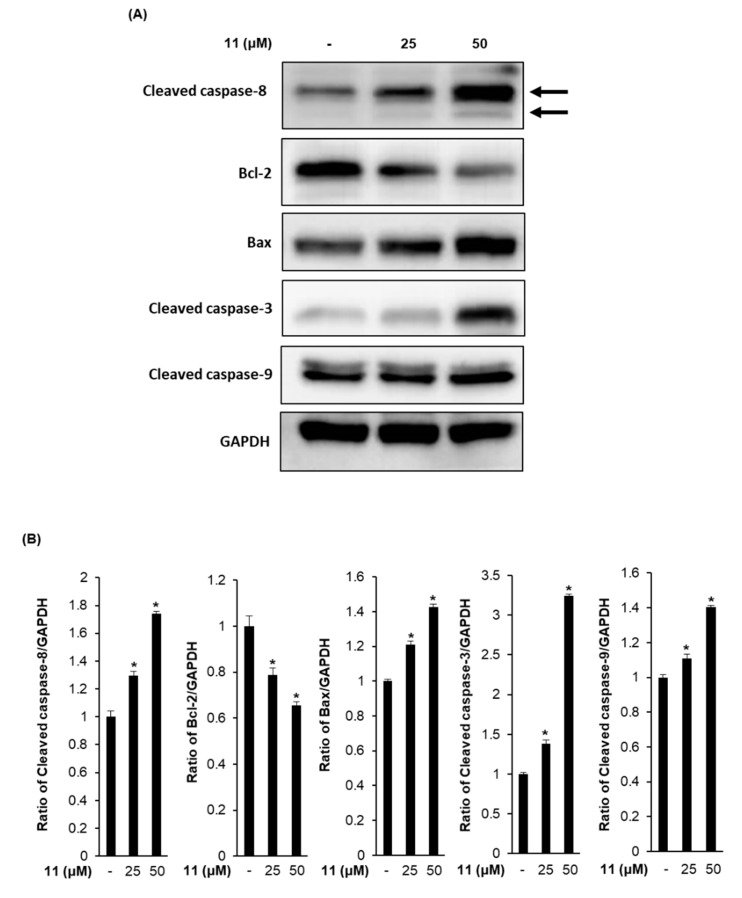
Effects of compound **11** on the expression of apoptosis-related proteins in A2780 human ovarian carcinoma cells. (**A**) A representative western blot is shown demonstrating the levels of cleaved caspase-8, Bcl-2, Bax, cleaved caspase-3, cleaved caspase-9, and glyceraldehyde-3-phosphate dehydrogenase (GAPDH) in A2780 cells treated with compound **11** (25 and 50 μM). GAPDH was used as an internal control. (**B**) Each bar graphs represents the densitometric quantification of western blot bands. * *p* < 0.05 compared to the values obtained for non-treated cells. Western blot assays were done in triplicate for each protein and were repeated at least three times.

Apoptosis is generally regulated by the interaction between components of the Bcl-2 family and caspase family of proteins [34]. Because results from staining with Hoechst 33342 and image-based cytometric assay provided evidence of apoptosis induction by treatment with compound **11**, we evaluated the expression of cleaved caspase-8, Bcl-2, Bax, cleaved caspase-3, and cleaved caspase-9 proteins by Western blot analysis (Figure 5). The densitometric quantification of Western blot bands is graphically presented in Figure 5B. In the analysis of the apoptotic initiator caspases, the treatment with compound **11** increased the expression of cleaved caspase-8 by 1.29 ± 0.02- and 1.73 ± 0.01-fold and expression of cleaved caspase-9 by 1.10 ± 0.01- and 1.40 ± 0.01-fold compared to the values obtained for non-treated cells when the concentration of compound **11** was increased from 25 µM to 50 µM, respectively. Caspase-8 and -9 have been known to play a crucial role in the apoptosis pathway as initiators [35]. Caspase-8 is responsible for mitochondria-independent apoptosis triggered by death receptors, which are cell surface receptors. On the other hand, caspase-9 is responsible for the mitochondria-dependent apoptosis triggered by the release of cytochrome c from mitochondria into the cytosol [36,37]. Activated caspase-8 and caspase-9 lead to downstream activation of the caspase-3 [34], acting as a common effector for the execution-phase of apoptosis pathway [38]. Our Western blot results for the apoptotic effector caspase showed that the expression level of cleaved caspase-3 was increased to 1.38 ± 0.04- and 3.23 ± 0.02-fold compared to the levels in non-treated cells when the concentration of compound **11** increased from 25 µM to 50 µM, respectively. These results suggest that compound **11** induces mitochondria-dependent and -independent apoptosis pathways through the activation of caspase-8 and -9. In addition, among the Bcl-2 family members, the treatment with compound **11** decreased the expression of anti-apoptotic Bcl-2 to 0.78 ± 0.02- and 0.65 ± 0.01-fold and increased the expression level of pro-apoptotic Bax to 1.20 ± 0.02- and 1.42 ± 0.01-fold compared to the levels in non-treated cells when the concentration of compound **11** increased from 25 µM to 50 µM, respectively. The mitochondria-independent apoptosis is mediated through the downregulation of anti-apoptotic Bcl-2 and upregulation of pro-apoptotic Bax. The anti-apoptotic Bcl-2 blocks mitochondrial permeability through the inhibition of Bax [39]. In this study, the results showed that expression levels of cleaved caspase-8, Bax, cleaved caspase-3, and cleaved caspase-9 increased in A2780 cells treated with compound **11**. Conversely, the levels of Bcl-2 decreased upon treatment with compound **11**.

Betulinic acid has been well known to inhibit proliferation and induce apoptosis in various cancer cell lines, including those of the breast, prostate, brain, colon, and leukemia [40,41,42,43,44]. It has recently been reported that betulinic acid induces apoptosis by stabilizing p53 and by downregulating the NF-κB pathway in human prostate cancer cells, irrespective of the androgen association [45]. Moreover, betulinic acid was reported to have in vivo anticancer activity in melanoma and prostate xenograft mouse models [42,46]. Additionally, in vivo studies particularly indicated that betulinic acid has a high safety margin without systemic side effects at the dose range tested [46]. According to recent biological studies on the anticancer effects of betulinic acid, it inhibits growth and induces apoptosis in RKO and SW480 colon cancer cells and inhibits tumor growth in athymic nude mice by proteasome-dependent and -independent downregulation of specificity protein (Sp) transcription factors, such as Sp1, Sp3, and Sp4 [47]. In addition, a study reported that betulinic acid decreases the growth of estrogen receptor (ER)-negative MDA-MB-231 breast cancer cells in vitro and in vivo by interactions with the microRNA-27a-ZBTB10-Sp-axis, causing increased cell death [48]. Furthermore, betulinic acid has been reported to exert cytotoxic activity against multidrug-resistant tumor cells by targeting autocrine motility factor receptor (AMFR), which is a ubiquitin E3-ligase cell surface glycoprotein that is known to play a role in metastasis, tumor progression, and recurrence [49]. Interestingly, a synergistic effect was observed in the anticancer activity when betulinic acid was used in combination with tumor necrosis factor-related apoptosis-inducing ligand (TRAIL) or ionizing radiation [50,51]. Therefore, together with these previously published studies, our data strongly suggest that betulinic acid can potentially be developed as a molecule of interest in cancer chemoprevention. Recently, it was reported that betulinic acid isolated from *Mimosa caesalpiniifolia* and its hexanoate derivative showed inhibition of cell proliferation against human ovarian carcinoma OVCAR-8 cell line [52,53]. Betulinic acid isolated from the stem bark extract of *Physocarpus intermedius* was shown to exhibit cytotoxicity against one of the ovarian cancer cell lines, SK-OV-3, in vitro [54]. The betulinic acid derivatives (methyl and benzyl esters, benzyl amide, and rhodamine B derivative of betulinic acid) were prepared by one research group, recently [55] and the betulinic acid derivatives showed significant cytotoxic activities in A2780 ovarian carcinoma cells [55]. In addition, antiproliferative activities for hydroxamates and carbamates of betulinic acid were examined against A2780 human ovarian carcinoma cells where most of the compounds were cytotoxic even in a low micro molar concentration [56]. Interestingly, it was demonstrated that 5-fluorouracil and betulinic acid combination has a synergistic effect in ovarian carcinoma treatment by inducing apoptosis through mitochondrial pathway and targeting the hedgehog signaling pathway [57]. Another interesting study revealed that betulinic acid can induce apoptosis in cisplatin-resistant ovarian cancer cells and Puma activation plays a critical role in mediating betulinic acid-induced apoptosis in cisplatin-resistant ovarian cancer cells [58].

## 4. Conclusions

Our study demonstrates the significant cytotoxic activity of betulinic acid isolated from the stem and stem bark of *C. walteri* in A2780 human ovarian cancer cells. Betulinic acid inhibited the proliferation of A2780 cells in a concentration-dependent manner. The apoptotic effects were further verified by a significant increase in the condensation of nuclear and apoptotic cell death in betulinic acid-treated A2780 cells. Betulinic acid upregulated the expression levels of cleaved caspase-8, -9, -3, and Bax and downregulated the expression of Bcl-2. These findings demonstrate that the apoptotic effect of betulinic acid was mediated through both extrinsic and intrinsic apoptotic pathways in A2780 cells. Our findings provide experimental evidence suggesting that betulinic acid can be a potential lead compound for therapeutic intervention in ovarian cancer.

## Supplementary Materials

The following are available online at https://www.mdpi.com/2218-273X/9/7/257/s1, ESI-MS, NMR spectral, and physical data of compounds **1–14** are available online at www.mdpi.com.
