# Supplementary material for: Betulinic Acid Suppresses Ovarian Cancer Cell Proliferation through Induction of Apoptosis
Source: Biomolecules. 2019 Jul 3;9(7):257. doi: 10.3390/biom9070257 (PMC6681197; doi:10.3390/biom9070257)
Supplement: Supplementary file 1 [file biomolecules-09-00257-s001.pdf]

# Supplementary Materials

# Betulinic Acid Suppresses Ovarian Cancer Cell Proliferation through Induction of Apoptosis

**Dahae Lee [1,+], Seoung Rak Lee [1,+], Ki Sung Kang [2], Yuri Ko [3], Changhyun Pang [4], Noriko Yamabe [2,*], and Ki Hyun Kim [1,*]**

[1] *School of Pharmacy, Sungkyunkwan University, Suwon 16419, Republic of Korea*

[2] *College of Korean Medicine, Gachon University, Seongnam 13120, Republic of Korea*

[3] *Department of Obstetrics and Gynecology, University of Ulsan, Asan Medical Center, Seoul 05505, Republic of Korea*

[4] *School of Chemical Engineering, Sungkyunkwan University, Suwon 16419, Republic of Korea*

[†] These authors contributed equally to this work.

**Norphytan (1).** Colorless gum; $^{1}$H NMR (500 MHz, $CDCl_3$): $\delta$ 0.78 (d, 6H, $J$ = 6.5 Hz), 0.80 (d, 12H, $J$ = 6.6 Hz), 0.97-1.03 (m, 4H), 1.04-1.09 (m, 4H), 1.13-1.25 (m, 8H), 1.27-1.33 (m, 2H), 1.43-1.48 (m, 4H); $^{13}$C NMR (125 MHz, $CDCl_3$): $\delta$ 19.7, 19.8, 22.6, 22.7, 24.5, 24.79, 24.80, 28.0, 32.78, 32.80, 37.3, 37.39, 37.41, 37.5, 39.4; ESI-MS $m/z$ 269 $[M+H]^+$.

**Leucophyllone (2).** White powder; $[\alpha]_D^{25}$ -98.5 ($c$ 0.35, MeOH); $^{1}$H NMR (500 MHz, $CDCl_3$): $\delta$ 0.82 (s, 3H), 0.88 (d, 3H, $J$ = 6.0 Hz), 1.01 (s, 6H), 1.05 (s, 3H), 1.12 (s, 3H), 1.26 (s, 6H), 3.16 (s, 3H), 5.30 (ddd, 1H, $J$ = 15.5, 8.0, 5.5 Hz); $^{13}$C NMR (125 MHz, $CDCl_3$): $\delta$ 12.7, 18.2, 18.4, 21.6, 21.9, 24.3, 24.5, 25.7, 26.2, 27.4, 28.0, 33.5, 34.0, 34.9 (x 2), 36.3, 38.5, 39.1, 43.4, 47.8, 48.4, 50.2, 51.1, 52.3, 52.5, 74.8, 117.8, 128.6, 136.6, 145.8, 216.9; ESI-MS $m/z$ 455 $[M+H]^+$.

**3-*O*-Acetylbetulin (3).** White powder; $[\alpha]_D^{25}$ +23.5 ($c$ 0.80, MeOH); $^{1}$H NMR (500 MHz, $CDCl_3$): $\delta$ 0.83 (s, 3H), 0.84 (s, 6H), 0.97 (s, 3H), 1.01 (s, 3H), 1.68 (s, 3H), 2.03 (s, 3H), 2.38 (m, 1H), 3.32 (d, 1H, $J$ = 10.5 Hz), 3.79 (d, 1H, $J$ = 10.5 Hz), 4.46 (m, 1H), 4.58 (s, 1H), 4.67 (d, 1H, $J$ = 2.0 Hz); $^{13}$C NMR (125 MHz, $CDCl_3$): $\delta$ 14.7, 15.9, 16.1, 16.5, 18.1, 19.0, 20.8, 21.3, 23.7, 25.1, 27.0, 27.9, 29.1, 29.7, 33.9, 34.1, 37.0, 37.2, 37.8, 38.3, 40.9, 42.7, 47.7, 47.8, 48.7, 50.3, 55.3, 60.5, 80.9, 109.7, 150.4, 171.0; ESI-MS $m/z$ 455 $[M+H]^+$.

**Betulinic acid methyl ester (4).** White powder; $[\alpha]_D^{25}$ +9.5 ($c$ 0.35, MeOH); $^{1}$H NMR (500 MHz, $CDCl_3$): $\delta$ 0.75 (s, 3H), 0.82 (s, 3H), 0.91 (s, 3H), 0.96 (s, 3H), 1.56 (s, 3H), 1.68 (s, 3H), 1.88 (m, 4H), 2.21 (m, 3H), 3.00 (m, 1H), 3.18 (m, 1H), 3.67 (s, 3H), 4.60 (s, 1H), 4.73 (d, 1H, $J$ = 2.0 Hz); $^{13}$C NMR (125 MHz, $CDCl_3$): $\delta$ 14.7, 15.3, 15.9, 16.1, 18.3, 19.3, 20.8, 25.5, 27.4, 27.9, 29.6, 30.6, 32.1, 34.3, 36.9, 37.1, 38.2, 38.7, 38.8, 40.6, 42.3, 46.9, 49.4, 50.5, 54.2, 55.3, 56.5, 78.9, 109.5, 150.5, 176.6; ESI-MS $m/z$ 471 $[M+H]^+$.

**6*β*-Hydroxysitostenone (5).** White powder; $[\alpha]_D^{25}$ +42.5 (*c* 0.65, MeOH); $^1$H NMR (500 MHz, $CDCl_3$): *δ* 0.74 (s, 3H), 0.81 (s, 3H, *J* = 6.5 Hz), 0.83 (d, 3H, *J* = 6.5 Hz), 0.85 (s, t, *J* = 7.0 Hz), 0.92 (d, 3H, *J* = 6.5 Hz), 1.38 (s, 3H), 4.35 (s, 1H), 5.81 (s, 1H); $^{13}$C NMR (125 MHz, $CDCl_3$): *δ* 11.9, 12.0, 18.7, 19.0, 19.5, 19.8, 20.9, 23.1, 24.1, 26.1, 28.2, 29.2, 29.7, 33.9, 34.5, 36.1, 36.6, 37.1, 38.5, 38.0, 42.5, 45.8, 53.6, 55.9, 56.0, 73.2, 126.3, 168.6, 200.4; ESI-MS *m/z* 429 $[M+H]^+$.

**Lupenone (6).** White powder; $[\alpha]_D^{25}$ +52.2 (*c* 0.25, MeOH); $^1$H NMR (500 MHz, $CDCl_3$): *δ* 0.76 (s, 3H), 0.82 (s, 3H), 0.87 (s, 3H), 0.90 (s, 3H), 0.92 (s, 3H), 0.97 (s, 3H), 1.10 (m, 2H), 1.69 (s, 3H), 2.27 – 2.36 (m, 1H), 4.61 (brs, 1H), 4.75 (brs, 1H); $^{13}$C NMR (125 MHz, $CDCl_3$): *δ* 14.7, 15.8, 16.2, 18.3, 19.3, 19.4, 20.9, 21.5, 25.6, 27.1, 29.5, 30.8, 32.0, 33.3, 34.0, 36.9, 37.1, 38.2, 39.5, 40.6, 42.4, 46.9, 47.2, 49.1, 50.3, 54.6, 55.0, 109.6, 150.6, 218.0; ESI-MS *m/z* 425 $[M+H]^+$.

**Methyl 3-*O*-acetylbetulinate (7).** White powder; $[\alpha]_D^{25}$ +34.8 (*c* 0.20, MeOH); $^1$H NMR (500 MHz, $CDCl_3$): *δ* 0.81 (s, 3H), 0.82 (s, 6H), 0.87 (s, 3H), 0.93 (s, 3H), 1.38 (s, 3H), 1.61 (s, 3H), 1.97 (s, 3H), 3.63 (s, 3H), 4.43 (m, 1H), 4.57 (brs, 1H), 4.70 (brs, 1H); $^{13}$C NMR (125 MHz, $CDCl_3$): *δ* 14.7, 15.9, 16.1, 16.5, 18.2, 19.4, 20.9, 21.1, 23.2, 25.4, 27.1, 27.9, 29.6, 29.8, 34.1, 34.3, 37.1, 37.8, 38.2, 38.4, 40.7, 42.4, 46.7, 47.0, 49.4, 50.4, 51.2, 56.5, 80.9, 109.6, 150.5, 170.9, 176.6; ESI-MS *m/z* 513 $[M+H]^+$.

**Phytone (8).** Colorless gum; $[\alpha]_D^{25}$ +3.4 (*c* 1.20, MeOH); $^1$H NMR (500 MHz, $CDCl_3$): *δ* 0.76–0.80 (m, 12H), 0.99–1.45 (m, 19H), 2.06 (s, 3H), 2.33 (t, 2H, *J* = 7.5 Hz); $^{13}$C NMR (125 MHz, $CDCl_3$): *δ* 20.0, 20.1, 21.8, 23.0, 23.1, 24.8, 25.2, 28.4, 30.2, 33.1, 33.2, 36.9, 37.6, 37.7, 37.8, 39.7, 44.5, 209.7; ESI-MS *m/z* 269 $[M+H]^+$.

**Lupeol (9).** White powder; $[\alpha]_D^{25}$ +28.4 (*c* 0.48, MeOH); $^1$H NMR (500 MHz, $CDCl_3$): $\delta$ 0.77, 0.80, 0.84, 0.95, 0.97, 1.03, 1.70 (s, each 3H), 2.38 (dt, 1H, *J* = 9.5, 4.5 Hz), 3.19 (dd, 1H, *J* = 11.5, 4.5 Hz, H-3), 4.57 (brs, 1H), 4.68 (brs, 1H); $^{13}$C NMR (125 MHz, $CDCl_3$): $\delta$ 14.9, 15.8, 16.3, 16.5, 18.4, 18.7, 19.7, 21.3, 25.5, 27.7, 27.8, 28.4 30.2, 34.6, 35.9, 37.5, 38.4, 39.1, 39.2, 40.4, 41.2, 43.2, 43.4, 48.3, 48.6, 50.7, 55.6, 79.3, 109.6, 151.1; ESI-MS *m/z* 427 $[M+H]^+$.

**Sitostenone (10).** White powder; $[\alpha]_D^{25}$ +74.5 (*c* 0.25, MeOH); $^1$H NMR (500 MHz, $CDCl_3$): $\delta$ 0.74 (s, 3H), 0.84 (d, 3H, *J* = 6.5 Hz), 0.86 (d, 3H, *J* = 6.5 Hz), 0.87 (t, 3H, *J* = 7.0 Hz), 0.94 (d, 3H, *J* = 6.5 Hz), 1.20 (s, 3H), 5.73 (s, 1H); $^{13}$C NMR (125 MHz, $CDCl_3$): $\delta$ 12.7, 12.9, 18.1, 19.7, 21.7, 21.8, 21.9, 23.8, 24.9, 26.7, 28.9, 29.8, 32.7, 33.7, 34.6, 34.7, 36.3, 36.4, 36.9, 39.3, 40.3, 43.1, 46.5, 54.5, 56.6, 56.7, 124.4, 172.6, 200.6; ESI-MS *m/z* 413 $[M+H]^+$.

**Betulinic acid (11).** White powder; $[\alpha]_D^{25}$ +7.5 (*c* 0.90, MeOH); $^1$H NMR (500 MHz, $CDCl_3$): $\delta$ 0.75 (s, 3H), 0.82 (s, 3H), 0.93 (s, 3H), 0.96 (s, 3H), 0.97 (s, 3H), 1.68 (s, 3H), 3.19 (dd, 1H, *J* = 10.0, 4.5 Hz), 4.60 (brs, 1H), 4.73 (brs, 1H); $^{13}$C NMR (125 MHz, $CDCl_3$): $\delta$ 14.7, 15.3, 16.0, 16.1, 18.3, 19.4, 20.8, 25.5, 27.4, 27.9, 29.7, 30.5, 32.1, 34.3, 37.0, 37.2, 38.4, 38.7, 38.8, 40.7, 42.4, 46.8, 49.2, 50.5, 55.3, 56.3, 78.9, 109.6, 150.3, 180.5; ESI-MS *m/z* 457 $[M+H]^+$.

**5*α*-Stigmast-3,6-dione (12).** White powder; $[\alpha]_D^{25}$ +14.6 (*c* 0.40, MeOH); $^1$H NMR (500 MHz, $CDCl_3$): $\delta$ 0.68 (s, 3H), 0.80 (d, 3H, *J* = 6.5 Hz), 0.82 (d, 3H, *J* = 6.5 Hz), 0.84 (t, 3H, *J* = 7.5 Hz), 0.92 (d, 3H, *J* = 6.5 Hz), 0.95 (s, 3H); $^{13}$C NMR (125 MHz, $CDCl_3$): $\delta$ 12.1, 12.2, 12.7, 18.8, 19.1, 20.0, 21.8, 23.2, 24.1, 26.1, 28.2, 29.2, 33.9, 36.2, 37.1, 37.5, 38.1, 38.2, 39.5, 41.4, 43.1, 45.9, 46.8, 53.6, 56.1, 56.7, 57.6, 209.6, 211.3; ESI-MS *m/z* 429 $[M+H]^+$.

**3*β*-Sitostanol (13).** White powder; $[\alpha]_D^{25}$ +22.4 (*c* 0.30, MeOH); $^1$H NMR (500 MHz, $CDCl_3$): *δ* 0.68 (s, 3H), 0.81 (d, 3H, *J* = 6.5 Hz), 0.83 (d, 3H, *J* = 6.5 Hz), 0.84 (t, 3H, *J* = 7.0 Hz), 0.92 (d, 3H, *J* = 6.5 Hz), 1.01 (s, 3H); ESI-MS *m/z* 417 $[M+H]^+$.

**6*α*-Hydroxy-*β*-sitostenone (14).** White powder; $[\alpha]_D^{25}$ +8.9 (*c* 0.30, MeOH); $^1$H NMR (500 MHz, $CDCl_3$): *δ* 0.71 (s, 3H), 1.18 (s, 3H), 0.92 (d, 3H, *J* = 6.5 Hz), 0.84 (d, 3H, *J* = 6.5 Hz), 0.81 (d, 3H, *J* = 6.5 Hz), 0.85 (t, 3H, *J*= 7.0 Hz), 4.33 (dd, 1H, *J*= 11.5, 4.5 Hz), 6.18 (s, 1H); $^{13}$C NMR (125 MHz, $CDCl_3$): *δ* 12.0, 12.1, 18.3, 18.7, 19.0, 19.8, 21.0, 23.1, 24.2, 26.1, 28.1, 29.2, 33.8, 33.9, 34.2, 36.1, 36.3, 39.0, 39.5, 41.5, 42.5, 45.9, 53.8, 55.6, 56.0, 68.6, 119.7, 158.7, 199.5; ESI-MS *m/z* 429 $[M+H]^+$.
